# Effect of Cementitious Capillary Crystalline Waterproofing Materials on the Mechanical and Impermeability Properties of Engineered Cementitious Composites with Microscopic Analysis

**DOI:** 10.3390/polym15041013

**Published:** 2023-02-17

**Authors:** Yan Tan, Ben Zhao, Jiangtao Yu, Henglin Xiao, Xiong Long, Jian Meng

**Affiliations:** 1 College of Civil Engineering, Architecture and Environment, Hubei University of Technology, Wuhan 430068, China; 2 School of Civil Engineering, Tongji University, Shanghai 200092, China

**Keywords:** cementitious capillary crystalline waterproofing material, engineered cementitious composites, mechanical properties, impermeability properties, chloride ion diffusion coefficient

## Abstract

Building structures are prone to cracking, leakage, and corrosion under complex loads and harsh marine environments, which seriously affect their durability performance. To design cementitious composites with excellent mechanical and impermeability properties, Engineered Cementitious Composites (ECCs) doped with ultrahigh molecular weight polyethylene short-cut fibers (PE-ECCs) were used as the reference group. Different types (XYPEX-type from Canada, SY1000-type from China) and doses (0%, 0.5%, 1.0%, 1.5%, 2.0%) of Cementitious Capillary Crystalline Waterproofing materials (CCCWs) were incorporated. The effect of CCCWs on the mechanical and impermeability properties of PE-ECCs, and the microscopic changes, were investigated to determine the best type of CCCW to use and the best amount of doping. The results showed that with increasing the CCCW dosage, the effects of both CCCWs on the mechanical and impermeability properties of PE-ECC increased and then decreased, and that the best mechanical and impermeability properties of PE-ECC were achieved when the CCCW dosing was 1.0%. The mechanical properties of the PE-ECC were more obviously improved by XYPEX-type CCCW, with a compressive strength of 53.8 MPa, flexural strength of 11.8 MPa, an ultimate tensile stress of 5.56 MPa, and an ultimate tensile strain of 7.53 MPa, which were 37.95%, 53.25%, 14.17%, and 21.65% higher than those of the reference group, respectively. The effects of the two CCCWs on impermeability were comparable. CCCW-PE-ECC(X1.0%) and CCCW-PE-ECC(S1.0%) showed the smallest permeation heights, 2.6 mm and 2.8 mm, respectively. The chloride ion diffusion coefficients of CCCW-PE-ECC(X1.0%) and CCCW-PE-ECC(S1.0%) exhibited the smallest values, 0.15 × 10^−12^ m^2^/s and 0.10 × 10^−12^ m^2^/s, respectively. Micromorphological tests showed that the particle size of the XYPEX-type CCCW was finer, and the intensity of the diffraction peaks of C-S-H and CaCO_3_ of PE-ECC increased after doping with two suitable doping amounts of CCCW. The pore structure was improved, the surface of the matrix was smoother, and the degree of erosion of hydration products on the fiber surface was reduced after chloride ion penetration. XYPEX-type CCCW demonstrated a more obvious improvement in the PE-ECC pore structure.

## 1. Introduction

Concrete is one of the main construction materials in modern civil engineering [1]. It is widely utilized in building coastal and marine infrastructures because of its good mechanical properties, simple construction, and low cost [2]. However, concrete, as a typical inorganic composite material, has hydrophilic and porous characteristics [3]. Additionally, concrete without reinforcement is prone to fracture under tensile and bending loads, thus providing an influx channel to aggressive ions under the combined actions of water, air, and temperature variations [4,5,6,7]. This greatly accelerates the decline in the mechanical properties and durability of concrete structures [8,9], affecting the safety and service lives of the structures [10].

Engineered Cementitious Composites (ECCs) are green, high-performance construction materials, designed based on micromechanics and fracture mechanics, that exhibit excellent tensile ductility at relatively low fiber content (typically ≤2% by volume fraction) [11,12]. The tensile strain of ECCs reaches at least 2%, which is more than 200 times that of ordinary concrete [13,14]. ECCs exhibit strain hardening and saturation cracking under increasing tensile forces, with crack widths typically ranging from 0.05 mm~0.1 mm [15,16,17,18]. Studies have shown that the permeability stabilizes within 3–4 days when the ECC fracture width is <60 μm, and takes 7–10 days, or even longer, to stabilize when the fracture width is >100 μm. A tightly cracked ECC can complete self-healing in a short period of time and can effectively prevent water molecules and corrosive ions from attacking the material [19]. The chloride ion diffusion coefficient of ECCs is only approximately 1/2 that of mortar and 10–35% that of concrete [20,21,22], and the chloride ion diffusion coefficient of ordinary cement mortar increases exponentially with preload deformation, while ECCs show a linear trend. This indicates that the chloride ion diffusion coefficient of ECCs is significantly lower than that of ordinary mortar under any preloading deformation [23]. It is generally believed that multiple fine cracks produced by an ECC under load can effectively limit the chloride ion penetration, but studies have shown that the resistance of an ECC to chloride ion intrusion depends mainly on the accumulated crack width rather than the maximum width [24,25]. Under prolonged exposure to chloride ions, ECCs still exhibited high durability, ductility, and multiple cracks, with a strain capacity of more than 2% [26,27,28].

Most ECCs are often prepared using polyvinyl alcohol (PVA) and polypropylene (PP) fibers, which typically achieve a 3% to 5% strain capacity [29]. Due to the presence of hydroxyl groups in the molecular chains, the chemical bonding of PVA fibers to the matrix is very strong, resulting in a large number of fibers that cannot be pulled out of the matrix but break directly. Oiling the surface of PVA fibers can significantly reduce the interfacial bond strength between the fibers and the matrix, to achieve the desired multiple-cracking behavior [30]. Compared with PVA fibers, PE fibers have a higher strength and elastic modulus. More importantly, unlike the hydrophilic nature of PVA, PE fibers are hydrophobic, which reduces the chemical bonding between the fibers and the substrate and makes PE fibers less likely to break during the pull-out process. These properties of PE fibers favor defect size tolerance and fiber bridging complementary energy [31,32], with great potential for the preparation of stronger ECCs [33,34,35]. With increasing PE fiber content, the tensile strain capacity, bending deformation capacity, and fiber bridging capacity increased significantly, while the first cracking stress, peak stress, flexural strength, and fiber bridging strength increased and then decreased, and the composites containing a 1.5% volume fraction of PE fibers had the highest peak stress, compressive strength, and excellent workability [32]. However, under the influence of extreme environments such as high corrosion, the fiber-matrix interface of PE-ECCs may be altered, thus affecting the durability performance of PE-ECCs. First, the PE-ECC is doped with a large number of polymer fibers, and as the PE fiber content increases, the interface area between the fibers and the matrix increases accordingly, providing more channels for ion transport. Second, during the hydration reaction of cement, a water film is easily formed around the polymer fibers, resulting in a high water-cement ratio and increased porosity in the area around the fibers, and the fine cracks of ECC still cannot effectively block the rapid invasion of water molecules and chloride ions under long-term immersion in seawater or extreme environments [36]. Therefore, enhancing the permeation resistance of PE-ECCs can help to improve their durability performance in extremely corrosive environments.

Cementitious Capillary Crystalline Waterproofing materials (CCCWs) are rigid waterproofing materials that offer the advantages of environmental protection, waterproofing, corrosion resistance, and self-healing [37,38]. K. Zheng’s studies have shown that the addition of CCCW promotes the hydration of cement and increases the condensation rate within a certain period of time, and that an appropriate amount of CCCW can greatly improve the mechanical and durability properties of cement-based materials [39]. When a CCCW is used as a surface coating, it has little effect on the compressive strength of the concrete, but when a CCCW is used as an admixture, it can greatly improve the compressive strength of the concrete [40]. This is because CCCWs can be uniformly dispersed in the structure during the mixing process, filling the pores through capillary crystallization reactions [41], which makes the structure more dense. Escoffres showed that under constant load, the incorporation of calcium is beneficial for restoring the mechanical properties of high-performance fiber-reinforced concrete, and the synergistic effect of fibers and calcium enables self-healing products to better bind to fibers [42]. Huang argues that when a structure cracks due to external conditions, internal fibers can play a crack resistance role, which helps CCCWs to better exert their performance [43]. When cracks appear in the concrete structure, the active chemicals in the CCCW penetrate into the concrete through the water, which acts as a carrier, which promotes a chemical reaction between the CCCW and cement hydration products and forms water-insoluble crystals, thereby blocking the pores and microcracks, effectively alleviating the penetration of chloride ions, prolonging the service life of the structure, and greatly reducing the maintenance costs caused by corrosion [44,45].

The improvement in structural impermeability imparted by CCCWs is related to the improvement in the internal pore structure, which depends on many factors, such as the CCCW content, composition, and environmental conditions [37]. Zheng et al. [39] showed that the total porosity of the structure decreased with increasing CCCW content. Azarsa et al. [41] demonstrated that CCCW increased the surface resistivity of concrete and reduced the chloride ion flux, thus improving the resistance of concrete to chloride ion penetration. CCCWs were more effective at improving the permeability resistance of the structural matrix as an admixture, compared to CCCW-coated specimens [37]. In general, the addition of a CCCW to concrete can improve the strain capacity of the material [46]. The CCCW has little effect on the material’s compatibility, and when the amount of the CCCW is appropriate, it can greatly improve the mechanical properties and durability of cementitious materials [37], while there are differences in the effects of different types of CCCWs on material properties. Addition of a CCCW may increase the types of self-healing products, change their morphology and quantity, and improve the properties of the structure [47]. The types and proportions of self-healing products vary depending on the location of the cracks, and the self-healing products of cracks on the surfaces of structures were mainly CaCO_3_ [48]. 

At present, there have been more studies on the role of CCCWs in ordinary concrete, but less research has been conducted on the impact of PE-ECC performance, especially the degree of impact on tensile and impermeability properties, which remains to be studied. Therefore, in this study, ECCs doped with ultrahigh molecular weight polyethylene short-cut fibers (PE-ECCs) were used as a benchmark group, and were mixed with different types and amounts of cementitious capillary crystalline waterproofing materials (CCCWs). Compressive tests, flexural tests, and tensile tests were used to test their mechanical properties, and the permeability height method and rapid chloride migration coefficient method were used to analyze their impermeabilities. Laser particle size distribution, XRD, MIP, and SEM measurements were used to examine the particle size distribution, phase composition, pore structure, and micromorphology of the materials, respectively, providing implications for the protection of structures and buildings under complex loading conditions and in extremely harsh marine environments.

## 2. Experimental Procedure

### 2.1. Materials and Mix Ratio

**Figure 1:**
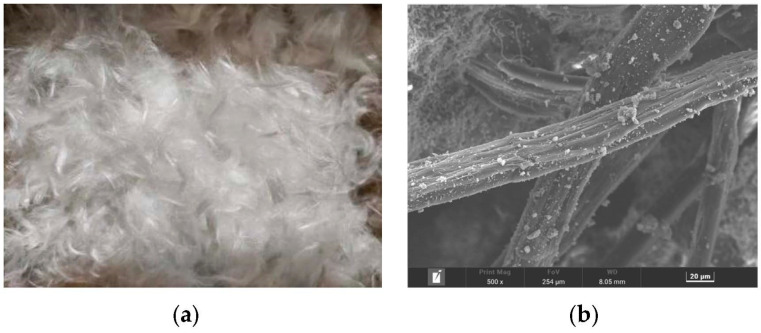
PE fiber: (**a**) Appearance; (**b**) SEM microtopography.

The raw materials prepared for casting specimens included PII 52.5 Portland cement, Class fly ash, fine sand, and polycarboxylic high-range water reducer (HRWR). The chemical compositions of the cement and fly ash, measured by XRF (X-ray fluorescence spectroscopy), are shown in Table 1. The fine sand was ultra-fine sand produced by Shanghai Fengxian Sand Factory, with specifications of 70~110 mesh and a maximum particle size of 0.21 mm. The water reducing agent was the high efficiency powder water reducing agent of polycarboxylic acid, produced by Shanghai Sanrui Company. Ultrahigh molecular weight polyethylene cropped fibers (PE) was selected as the reinforcement. The appearance and microscopic morphology of the PE fibers are shown in Figure 1a,b, the fibers have a disordered distribution. The fiber length was 12 mm, fiber diameter was 25 μm, and the density and elastic modulus of the fiber were 0.97 g/cm^3^ and 116 GPa, respectively. The volume fraction of PE fibers in the mix was 1.5%.

**Figure 2:**
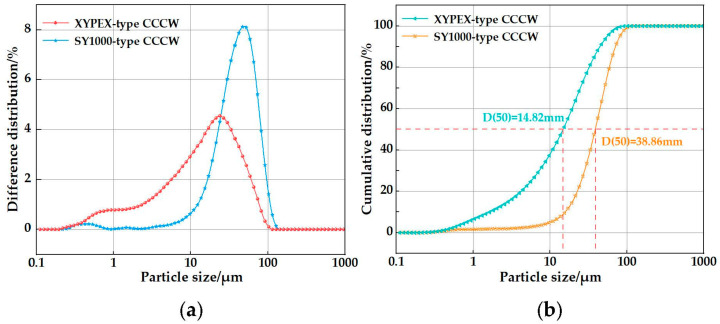
Particle size distribution of the XYPEX-type and SY1000-type CCCWs: (**a**) Difference particle size distributions; (**b**) Cumulative particle size distributions.

XYPEX-type CCCW from Canada, and SY1000-type CCCW developed by Yu Jianying’s team from the Wuhan University of Technology in China, were selected. The particle size distributions of the XYPEX-type CCCW and SY1000-type CCCW are presented in Figure 2. For the XYPEX-type CCCW, most of the particle sizes were less than 102.2 μm. Fifty percent and 90% of the particles had sizes of less than 14.82 μm and 44.68 μm, respectively. The most probable distribution of the particle size was 23.96 μm. For SY1000, most of the particle sizes were less than 114.6 μm. Fifty percent and 90% of the particles had sizes less than 38.86 μm and 70.83 μm, respectively. The most probable distribution of the particle size was 46.86 μm. Clearly, the XYPEX-type CCCW is more fine than the SY1000-type.

**Figure 3:**
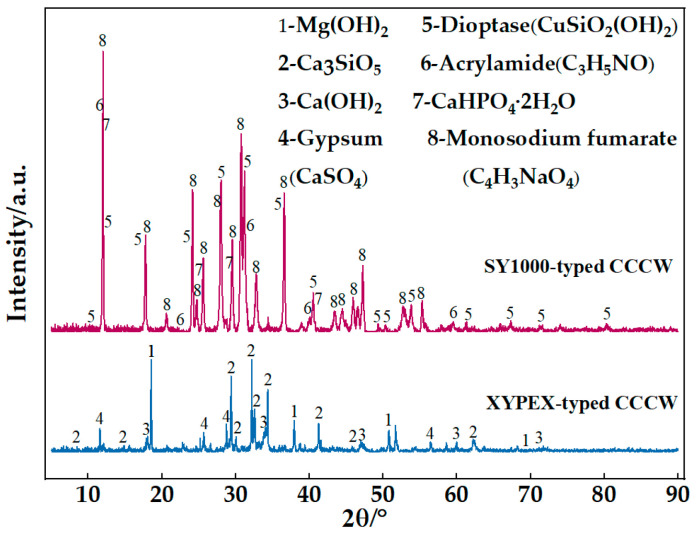
XRD patterns of PE-ECC, CCCW-PE-ECC (X1.0%), and CCCW-PE-ECC (S1.0%).

After 28 days of curing, X-ray diffraction analysis (D8, Advance, Bruker) was performed to identify the chemical compositions of the two different kinds of CCCWs, the phase composition (Figure 3).

The main components of the XYPEX-type CCCW included Ca(OH)_2_, Mg(OH)_2_, Ca_3_SiO_5_, and CaSO_4_. Ca_3_SiO_5_ reacted with H_2_O to produce calcium silicate hydrate (C-S-H) gel, which can effectively fill the cracks and pores in cement-based materials. As the compensators of Ca^2+^, Ca(OH)_2_ and CaSO_4_ provide a large amount of Ca^2+^ as Ca^2+^ complexants, and Ca^2+^ complexants can reduce the activation energy of hydration products reacting with calcium ions. When reaching the area where the cement gel is enriched, due to the different solubilities and stabilities of the products, the anions in the complexing agent will be replaced by silicate and aluminate ions. This can generate a large number of stable insoluble Ca^2+^ complexes in the water environment, promote the healing of cracks in the matrix material, and effectively resist the erosion of chloride ions.

The SY1000-type CCCW mainly contained clairvorite (CuSiO_2_(OH)_2_), acrylamide (C_3_H_5_NO), and dicalcium hydrogen phosphate (CaHPO_4_·2H_2_O), and sodium fumarate (C_4_H_3_NaO_4_). CaHPO_4_·2H_2_O was deemed an enhancer of Ca^2+^ as it can provide large quantities of Ca^2+^ in the water. Therefore, more Ca^2+^ complexes tended to be generated, which was conducive to the filling of cracks and pores. C_3_H_5_NO can not only improve the physical properties of synthetic fibers but also has the ability to prevent corrosion. In combination with C_4_H_3_NaO_4_, a new type of antiseptic, it obviously promoted the anticorrosion of the materials. The two advantages, i.e., the favorable formation of Ca^2+^ complexes and anticorrosion effect, demonstrated the better chloride ion resistance of the SY1000-type CCCW.

The mass fractions of CCCW used were set as 0%, 0.5%, 1.0%, 1.5%, and 2.0% of the cementitious material. The mix proportions are listed in Table 2.

### 2.2. Experimental Programs

An LJ-XLG50E blender was used to mix the raw materials. First, 80% of the water was added to the mixer, and then all the cement, sand, fly ash, and HRWR were added and mixed for 1 min. The remaining 20% of the water was added during the mixing process. After mixing for 3–5 min, PE fibers were gradually added and stirred for 5 min until the fibers were well dispersed. Finally, the fresh PE-ECC mortar was placed into the mold. All the specimens were demolded after one day and cured in a constant temperature and humidity container for 28 days.

**Figure 4:**
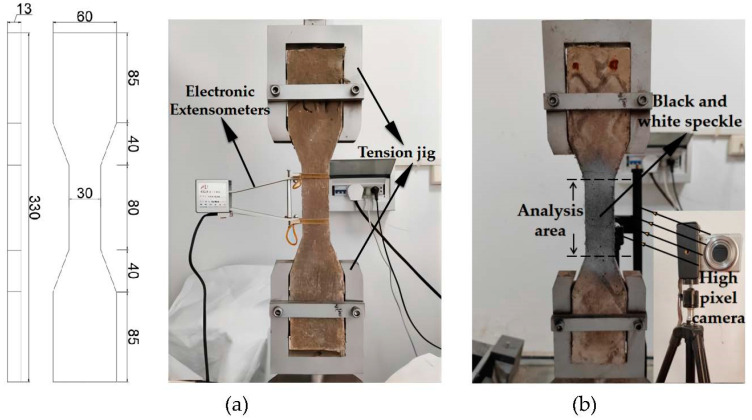
(**a**) Dog-bone specimen dimensions and test device; (**b**) DIC test.

The compressive strength and flexural strength were tested using a DYE-300S hydraulic servo loading system. The dimensions of the specimens used for testing were 70.7 mm × 70.7 mm × 70.7 mm and 40 mm × 40 mm × 160 mm, respectively. The tensile tests were conducted using a WDW-100C electronic servo loading system, and the loading rate was set to 1.5 mm/min. As shown in Figure 4a, electronic extensometers were used to measure the elongation.

The crack morphology and distribution were tested by digital image correlation (DIC). First, black and white paint was sprayed on the specimen to form a random speckle pattern, as shown in Figure 4b. Then, high-resolution photographs of the target area on the specimen surface were taken every 10 s during uniaxial tensile loading, and finally, the photographs were imported into the MATLAB software to calculate the local strain by tracking the movement of pixels in small areas before and after deformation, to generate strain maps at different loading stages.

**Figure 5:**
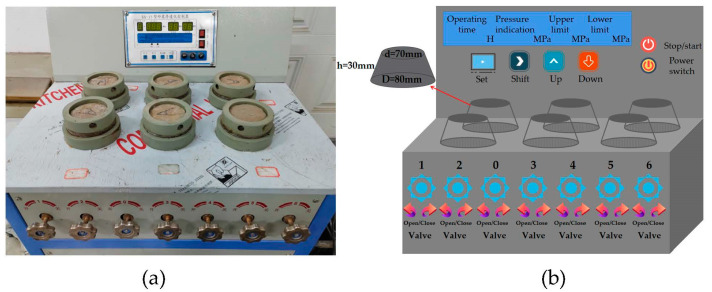
(**a**) SS-15 Mortar penetrometer; (**b**) Schematic diagram.

The permeability test was performed using the permeability height method, via an SS-15 mortar permeability meter, test equipment, and the specific specimen size, as shown in Figure 5a,b. After the test began, the water pressure was maintained at a constant (1.2 ± 0.05) MPa and maintained for 24 h. At the end of the test the specimens were split into two halves, using a waterproof pen to trace the watermarks and a steel ruler along the watermarks at equal intervals to measure 10 measuring points. Finally, the average value was used as the water penetration height of the group of specimens.

**Figure 6:**
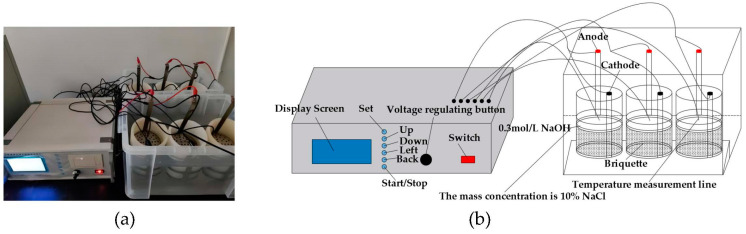
(**a**) Anti-chloride ion penetration test; (**b**) Schematic diagram.

**Figure 7:**
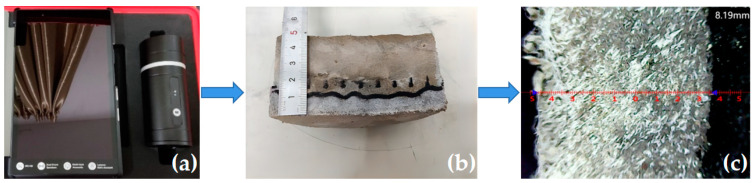
Chloride ion penetration depth measurement: (**a**) KS-105 wireless electronic meter; (**b**) Penetration depth of chloride ions; (**c**) Penetration depth test.

The chloride ion penetration test was performed based on the rapid chloride-ion migration coefficient method (RCM) stipulated in GB/T50082-2009 [49]. The diameter and height of the cylinder specimens were 100 mm and 50 mm, respectively. Before the test, a water saturation machine was used to vacuum water the specimens for (18 ± 2) h. Approximately 300 mL of NaOH solution, with a concentration of 0.3 mol/L, was injected into the rubber sleeve, and 12 L of NaCl solution, with a mass concentration of 10%, was injected into the cathode test tank and made flush with the liquid level of the NaOH solution in the rubber sleeve, as shown in the test diagram and schematic diagram in Figure 6a,b. After the experiment, the cylinders were cut into halves along the diameter and then sprayed with 0.1 mol/l AgNO_3_ solutions onto the cut sections. After that, the penetration depth of chloride ions was measured by using a KS-105 wireless electronic meter, as shown in Figure 7a–c.

(1) $$D_{\mathrm{RCM}}=\frac{0.0239\;\times \;\left(273\;+\;T\right)L}{\left(U\;-\;2\right)t}{(X}_{d}\;-\;0.0238\sqrt{\frac{\left(273\;+\;T\right){\mathrm{LX}}_{d}}{U\;-\;2}})$$

*D*_RCM_—Nonstationary chloride ion mobility coefficient; *U*—absolute value of the voltage used (V); *T*—average of the initial and ending temperatures of the anode solution (°C); *L*—specimen thickness (mm); *X*_d_—average value of chloride ion penetration depth (mm); and *t*—duration of the test (h).

Furthermore, a BT-9300S laser particle size distribution instrument was used to detect the particle size of the two types of CCCWs, and X-ray diffractometry (XRD; Brooke D8 advanced X-ray diffractometer) was used to test the phase composition of the two types of CCCWs, as well as the hydration products of PE-ECC and CCCW-PE-ECC. The PE-ECC and CCCW-PE-ECC pore distributions were determined using an AutoPore Iv 9520 mercury pressure meter. Scanning electron microscopy (SEM) was used to observe the micromorphologies of the PE-ECC and CCCW-PE-ECC specimens before and after chloride ion penetration.

## 3. Results and Discussion

### 3.1. Mechanical Properties

**Figure 8:**
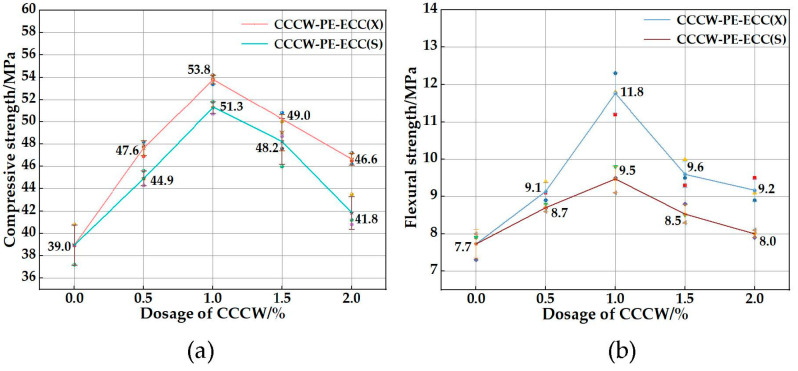
(**a**) Compressive strength; (**b**) Flexural strength.

The compressive strength and flexural strength of CCCW-PE-ECC(X) and CCCW-PE-ECC(S) are shown in Figure 8a,b. The compressive strength and flexural strength of PE-ECC were increased to a certain extent after mixing two kinds of CCCW separately, because the CCCW contains not only active substances but also a large amount of Ca^2+^ and SiO_3_^2−^, which can generate more calcium carbonate (CaCO_3_) and calcium silicate hydrate (C-S-H) and other gel products while catalyzing the hydration reaction of cement, filling the pores and cracks. The density of the matrix was improved. The compressive and flexural strengths of CCCW-PE-ECC(X) and CCCW-PE-ECC(S) were enhanced and then weakened with increased CCCW doping. The compressive strengths of CCCW-PE-ECC(X1.0%) and CCCW-PE-ECC(S1.0%) reached maximum values of 53.8 Mpa and 51.3 Mpa, respectively, which are 37.95% and 31.54% higher than those of the PE-ECC. The flexural strengths of CCCW-PE-ECC(X1.0%) and CCCW-PE-ECC(S1.0%) were 11.8 Mpa and 9.5 Mpa, respectively, which are 53.25% and 23.38% higher than those of the PE-ECC. Moreover, the effect of the XYPEX-type CCCW on the compressive and flexural strengths is more obvious. This may be because the particle size of the XYPEX-type is finer than that of the SY1000-type, and the hydration reaction of cement is more efficient, which results in a denser microstructure and matrix-fiber interface.

**Figure 9:**
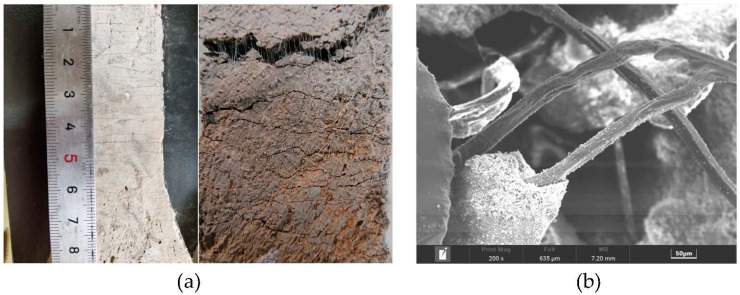
(**a**) Tensile crack distribution; (**b**) Microscopic morphology at the fracture.

As shown in Figure 9a, under uniaxial tension, the PE-ECC showed excellent crack control ability. Different from the brittle failure that occurred in ordinary concrete, the failure modes of PE-ECC were ductile failures, extending from one single crack to multiple fine cracks. All the PE-ECCs demonstrated tensile strain-hardening behaviors under tension, indicating that PE-ECC can still bear a higher load with a continuous increase in strain. Additionally, the cracking at the first crack point gradually developed into multiple cracking in the whole range. The microscopic morphology of the matrix material and PE fibers following tensile damage is shown in Figure 9b, where the PE fibers are pulled out instead of being pulled off. These properties of PE fibers favor defect size tolerance and fiber bridging complementary energy. This indicates that the PE fibers can withstand greater deformation and that the material ductility was significantly enhanced.

**Figure 10:**
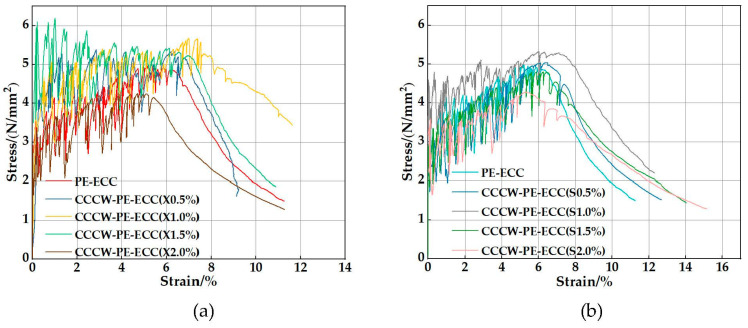
Stress-strain curves of (**a**) CCCW-PE-ECC(X) and (**b**) CCCW-PE-ECC(S).

**Figure 11:**
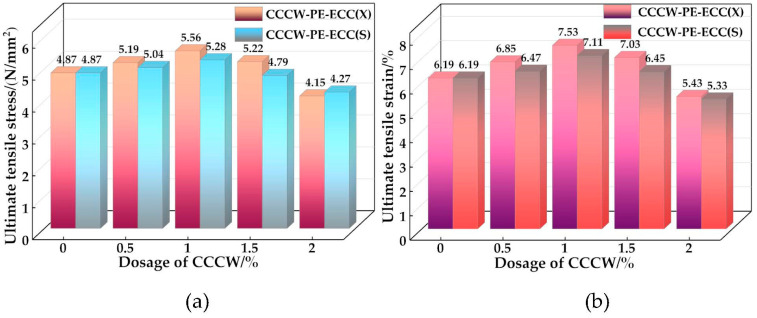
Ultimate tensile stress and ultimate tensile strain of CCCW-PE-ECC(X) and CCCW-PE-ECC(S): (**a**) Ultimate tensile stress; (**b**) Ultimate tensile strain.

The stress-strain curves of CCCW-PE-ECC(X) and CCCW-PE-ECC(S) are shown in Figure 10a,b. The stress-strain curves can be roughly divided into three stages: elastic stage, crack stable development stage, and local crack expansion fiber pull-out stage. From the beginning of loading to the appearance of the first crack, the stress and strain increase linearly, and in the crack stable development stage the strain hardening phenomenon is presented. Finally, in the local crack expansion fiber pull-out stage, no new cracks are generated, and the local cracks keep expanding until the maximum stress appears and the specimen is pulled out. The ultimate tensile stress and ultimate tensile strain are shown in Figure 11a,b. With increasing CCCW doping, the ultimate tensile stress and ultimate tensile strain of CCCW-PE-ECC(X) and CCCW-PE-ECC(S) show an increasing and then decreasing trend. The ultimate tensile stress and ultimate tensile strain of CCCW-PE-ECC(X1.0%) and CCCW-PE-ECC(S1.0%) reach ultimate tensile stress values of 5.56 N/mm^2^ and 5.28 N/mm^2^, which are 14.17% and 8.42% increases, respectively, compared with the reference group. The ultimate tensile strains were 7.53% and 7.11%, which are 21.65% and 14.86% increases, respectively, compared with the reference group. The ultimate tensile stress and ultimate tensile strain of CCCW-PE-ECC(X2.0%) and CCCW-PE-ECC(S2.0%) exhibit the minimum values. The ultimate tensile stress values are 4.15 N/mm^2^ and 4.27 N/mm^2^, which are 14.78% and 12.32% lower than the benchmark group, and the ultimate tensile strains are 5.43% and 5.33%, which are 12.28% and 13.89% lower than the benchmark group, respectively. This shows that the tensile properties of the PE-ECC are improved to a certain extent when the right amount of CCCW is incorporated, while an excessive amount of CCCW reduces the tensile properties of the PE-ECC. When an appropriate amount of CCCW was added, the generated gel products effectively filled the internal pores and cracks of the matrix, enhanced its compactness, promoted the bridging properties between the matrix and fibers, and thus enhanced its tensile properties. In contrast, excessive CCCW led to the generation of excessive hydration products, which caused the internal volume of the matrix to expand to a certain extent and destroyed the balance of mechanical interaction among the fibers, matrix, and composite interface in PE-ECC. The balance of the mechanical interaction among the three in the PE-ECC led to the weakening of its tensile properties.

**Figure 12:**
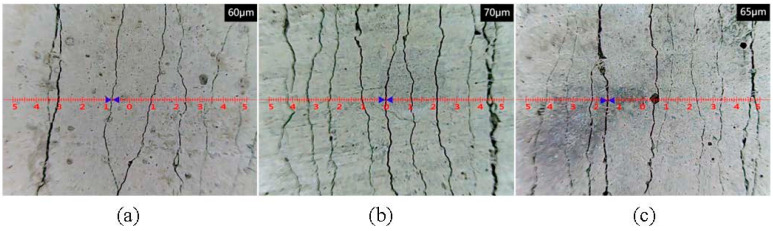
Crack distribution after stretching of (**a**) PE-ECC, (**b**) CCCW-PE-ECC (X1.0%), and (**c**) CCCW-PE-ECC (S1.0%).

The regulation of crack width has a significant influence on improving the mechanical properties and durability of cementitious materials. The standard requires that for structural components exposed to an adverse environment, or specially designed for anti-seepage, the crack width should be less than 200 μm [50]. As shown in Figure 12a–c, uniformly distributed cracks were narrowed when PE-ECC, CCCW-PE-ECC(X), and CCCW-PE-ECC(S) were subjected to tension, and the corresponding average crack widths were 60 μm, 70 μm, 65 μm, respectively. Evidently, all the crack widths were far less than the specification requirements [50], indicating that the aforementioned ECCs had acceptable durability. With the addition of CCCW, the crack spacing of PE-ECC became wider. Additionally, the number of cracks within the 80 mm gauge length of dog-bone-shaped specimens cast with PE-ECC, CCCW-PE-ECC(X), and CCCW-PE-ECC(S) was 42, 63, and 56, respectively. This demonstrates that the appropriate addition of CCCW caused an increase in the crack width and the number of cracks, thus enhancing the tensile deformation capacity.

**Figure 13:**
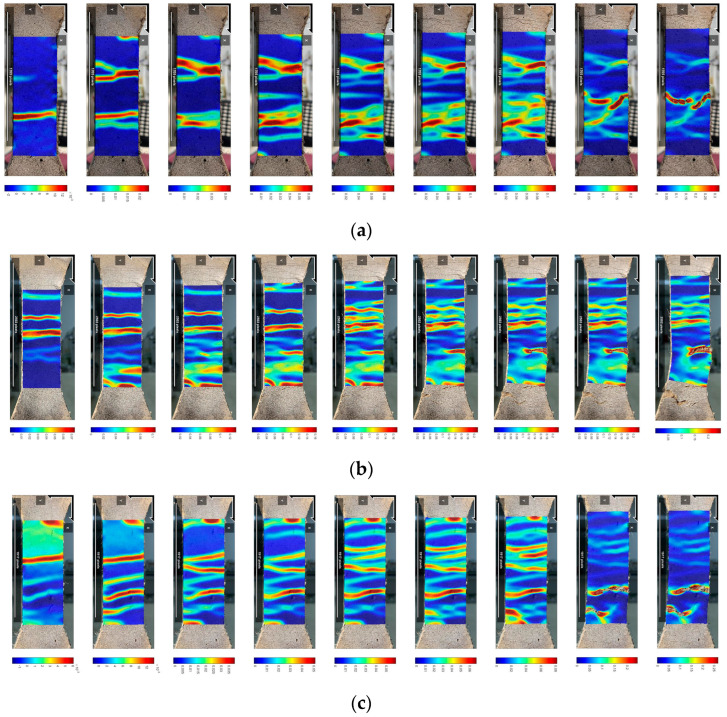
Strain clouds of (**a**) PE-ECC, (**b**) CCCW-PE-ECC(X1.0%) and (**c**) CCCW-PE-ECC(S1.0%).

The crack morphology and distribution were tested by digital image correlation (DIC). As shown in Figure 13a–c, as the load increases from zero, the tensile stress inside the cement matrix composite increases linearly until it reaches the elastic limit state. When the stress intensity factor is equal to the local matrix fracture toughness, the maximum crack internal defect, or weak zone, causes microcrack extension. When the tensile stress reaches the initial cracking strength, flattened matrix microcracks expand almost instantaneously on the specimen surface. After satisfying the multiple crack energy criterion, the cracks on the specimen surface expand in a steady-state flat crack expansion mode. A slight decrease in tensile stress occurs due to the sudden loss of the load transfer capability of the matrix. However, the fiber bridging stress does not exceed the matrix cracking stress, and as the tensile strain increases, the tensile stress not only recovers but exceeds the initial crack strength. The tensile stress continues to increase until another microcrack appears at the next largest defect. New flattened cracks continue to form and expand until the tensile stress at the weakest cross section of all cracked sections of the specimen exceeds the fiber bridging capacity. Eventually, the damage is concentrated at the weakest part of the bridging action. Compared with PE-ECC, CCCW-PE-ECC(X1.0%) and CCCW-PE-ECC(S1.0%) have a more uniform distribution of the main cracks throughout the middle region. Numerous fine saturated microcracks can be formed between the main cracks, the total deformation in the intermediate region increases, and the ductility is enhanced. Thus, the tensile deformation capacity of the material has been improved.

### 3.2. Water Seepage Resistance

**Figure 14:**
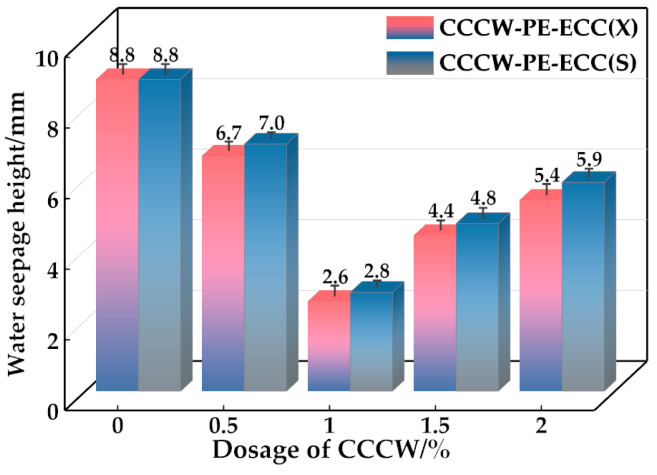
Water seepage height of CCCW-PE-ECC(X) and CCCW-PE-ECC(S).

The water seepage heights of CCCW-PE-ECC(X) and CCCW-PE-ECC(S) are shown in Figure 14. With increasing CCCW doping, the water seepage heights of both CCCW-PE-ECC(X) and CCCW-PE-ECC(S) show a trend of first decreasing and then increasing. CCCW-PE-ECC(X1.0%) and CCCW-PE-ECC(S1.0%) show the smallest permeation heights, 2.6 mm and 2.8 mm, respectively, which are 69.77% and 68.18% lower than those of the baseline group. Both CCCWs improve the impermeability of the PE-ECC to a similar extent. A CCCW can act synergistically with the PE fibers to promote the compactness of the matrix material. The principle is that when the cementitious material is in a dry environment, the active substance in CCCW is in a dormant state inside it, and when it is in a water environment or a wet state, the water pressure prompts the water molecules to penetrate into the pores and cracks inside the cementitious material with the active substance, and the active substance and Ca^2+^ in the material undergo a precipitation reaction to produce insoluble Ca^2+^ precipitate. At this time, part of the precipitate attaches to the surface of the PE fibers, which strengthens the bond between the fibers and the matrix, and the other part fills the pores and cracks, blocks the transmission of water molecules inside the matrix, and improves the water seepage resistance of the cementitious materials. However, excessive CCCW leads to an increase in hydration products and a certain expansion of the matrix volume, resulting in an increase in pores and cracks and a slight decrease in the water seepage resistance.

### 3.3. Anti-Chloride Ion Penetration Performance

**Figure 15:**
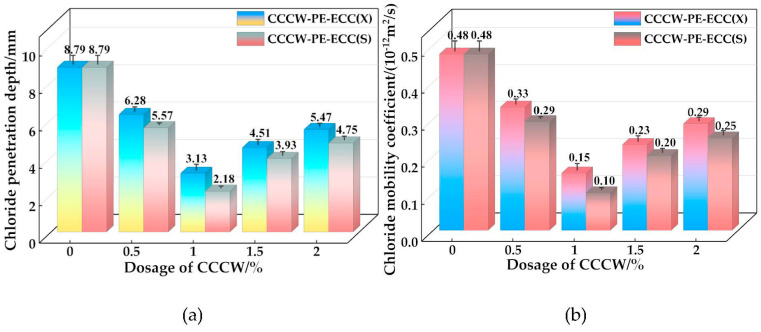
(**a**) Chloride ion permeation height; (**b**) Chloride ion mobility coefficient.

The chloride ion permeation heights and chloride ion diffusion coefficients of CCCW-PE-ECC(X) and CCCW-PE-ECC(S) are shown in Figure 15a,b, respectively. With increasing CCCW doping, the chloride ion permeation heights of CCCW-PE-ECC(X) and CCCW-PE-ECC(S) show a decreasing and then increasing trend, and the chloride ion permeation heights of CCCW-PE-ECC(X1.0%) and CCCW-PE-ECC(S1.0%) show minimum chloride ion permeation heights of 3.13 mm and 2.18 mm, respectively, which are 64.39% and 75.20% lower than the baseline group. The chloride ion diffusion coefficients shows a decreasing and then increasing trend, and CCCW-PE-ECC(X1.0%) and CCCW-PE-ECC(S1.0%) exhibit the smallest values, 0.15 × 10^−12^ m^2^/s and 0.10 × 10^−12^ m^2^/s, respectively, which are 68.75% and 79.17% lower than those of the reference group.

The anti-chloride ion permeation performance of PE-ECC is enhanced to a certain extent by incorporating an appropriate amount of CCCW, while the incorporation of excessive CCCW leads to a specific decrease in the anti-chloride ion permeation performance. This is because the incorporation of an appropriate amount of CCCW can effectively promote the hydration reaction of cement, generating a more suitable content of hydration products to fill the pores, and blocking the transmission of chloride ions inside the matrix, effectively inhibiting the erosion of chloride ions and enhancing the resistance to chloride ion penetration. However, an excessive amount of the CCCW will lead to the generation of excessive hydration products, resulting in matrix cracking damage, some pores, and cracks, and if there is an excessive amount of calcium alumina (AFT) in the hydration products, then excessive volume expansion occurs, which can have a negative impact on the cement matrix, thus leading to a decline in the chloride ion penetration resistance. The combined results of the chloride ion permeation heights and chloride ion diffusion coefficients show that the SY1000-type CCCW had better impermeability resistance than the XYPEX-type CCCW. This may be because the SY1000-type CCCW contains some anticorrosive substances, such as acrylamide (C_3_H_5_NO) and sodium fumarate (C_4_H_3_NaO_4_), which can effectively neutralize Cl^−^, reduce the erosion of Cl^−^ on the matrix material, and effectively improve the material’s resistance to chloride ion penetration.

## 4. Microscopic Analysis

### 4.1. Phase Composition Testing and Analysis

After 28 days of curing, X-ray diffraction analysis (D8, Advance, Bruker) was performed to identify the chemical compositions of the hydration products with PE-ECC, CCCW-PE-ECC(X1.0%), and CCCW-PE-ECC(S1.0%).

**Figure 16:**
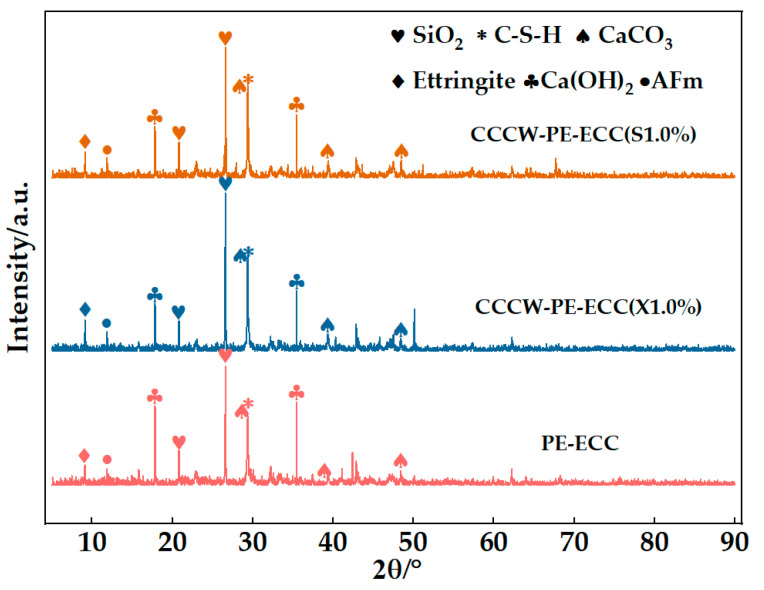
XRD patterns of PE-ECC, CCCW-PE-ECC(X1.0%) and CCCW-PE-ECC(S1.0%).

According to Figure 16, the same hydration products, including SiO_2_, C-S-H, CaCO_3_, and ettringite, exist in PE-ECC, CCCW-PE-ECC(X1.0%), and CCCW-PE-ECC(S1.0%), in which SiO_2_ accounted for the largest proportions and the intensity diffraction peaks of C-S-H, CaCO_3_, and ettringite in the CCCW-PE-ECC(X1.0%) and CCCW-PE-ECC(S1.0%) are higher than those in the reference PE-ECC. CaCO_3_ serves as a nucleation matrix, decreasing the potential barrier of nucleation and thus accelerating cement hydration. C-S-H gel is prone to forming in pores, which can refine the microstructures and then increase the compactness. Despite the existence of the volume expansion effect, the formation of ettringite is not necessarily regarded negatively. The expansion can somehow be helpful in promoting strength development at an early stage, compensating for shrinkage. It is safe to say that the addition of CCCW could promote the hydration reaction of the matrix, due to differences in concentration and pressure, the active substances in the CCCW can penetrate into a matrix with water through microcracks and then react with free lime and oxides in the pores to form an insoluble crystalline substance. In a dry environment, the active chemical precipitates out into its solid form and remains dormant, and when microcracks appear in the matrix, the water re-excites the activity of the active chemical, causing it to continue to diffuse and react until the crack is filled and compacted.

The peak intensity diffraction of Ca(OH)_2_ in CCCW-PE-ECC(X1.0%) and CCCW-PE-ECC(S1.0%) was reduced compared to PE-ECC because the active substance in CCCW was able to consume the Ca(OH)_2_ present in the cement base. This plays an important role in terms of the durability. The chemical reaction to reduce the Ca^2+^ content in substances is beneficial for the strength and compactness of the material, thus improving the overall strength and impermeability of the material.

### 4.2. Pore Structure Testing and Analysis

CCCWs can optimize the pore structure in EECs by improving their structural compactness [43]. The data obtained for PE-ECC, CCCW-PE-ECC(X1.0%), and CCCW-PE-ECC(S1.0%) are shown in Table 3, where the total pore volume, total pore area, and average pore size of CCCW-PE-ECC(X1.0%) and CCCW-PE-ECC(S1.0%) were decreased, while the bulk density and apparent density were increased, leading to a decrease in permeability. It is implied that the structure of the PE-ECC was more compact after incorporating the CCCW.

**Figure 17:**
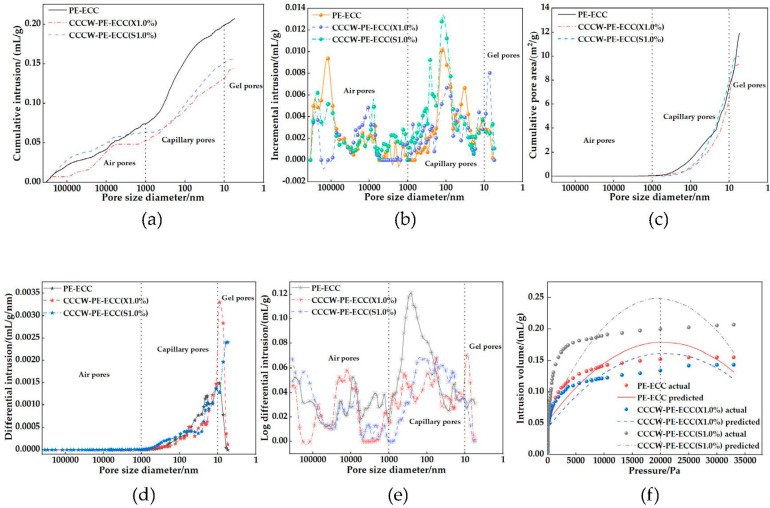
(**a**) Total pore volume vs. pore size; (**b**) Amount of variation in the pore; (**c**) The total pore area varies with pore size; (**d**) Pore size density distribution function; (**e**) Distribution map of the pore structure; (**f**) The total pore volume changes with pressure.

According to pore size, pores in concrete can be divided into air pores (d > 1 μm), capillaries (10 nm~1 μm), and gel pores (d < 10 nm) [51,52]. As shown in Figure 17a, the total pore volume of CCCW-PE-ECC(X1.0%) was 0.14 mL/g, and the total pore volume of CCCW-PE-ECC(S1.0%) was 0.16 mL/g. While the total pore volume of PE-ECC was 0.21 mL/g, compared with PE-ECC, the total pore volumes of CCCW-PE-ECC(X1.0%) and CCCW-PE-ECC(S1.0%) were reduced by 33.33% and 23.81%, respectively, and the capillary pores and gel pores were greatly reduced, indicating that the internal pore structure of PE-ECC was effectively improved after incorporation of the CCCW, the pores were reduced and the structure was more compact. The pore volume varies with pore size, as shown in Figure 17b. The PE-ECC pore volume varies greatly in the range of stomatal pores, the pore volume of CCCW-PE-ECC(S1.0%) changes greatly in the pore range, and CCCW-PE-ECC(X1.0%) varies greatly in the gel pore range, indicating that the CCCW-PE-ECC(X1.0%) pores were more uniformly distributed in the range of stomatal pores and pores, and the overall pore volume was small. The trend of total pore area with pore size is shown in Figure 17c. When the diameter was greater than 1000 nm, the pore area was almost zero, and the proportion of large pores was very small, indicating that the pores were mainly composed of pores and gel pores. The total pore area of PE-ECC was 11.90 m^2^/g, and the total pore areas of CCCW-PE-ECC(X1.0%) and CCCW-PE-ECC(S1.0%) were 9.43 m^2^/g and 9.98 m^2^/g, respectively, which were 20.76% and 16.13% lower than that of PE-ECC. This indicates that the number of PE-ECC pores decreased after incorporation of the CCCW. The pore density distribution function is shown in Figure 17d. The physical significance of the pore density distribution function is to divide the entire aperture distribution range into several pores of 1 nm. If there is a hole with a certain nanometer size, then the pore capacity value of this hole is expressed in ordinate coordinates. When the pore sizes of PE-ECC, CCCW-PE-ECC(X1.0%), and CCCW-PE-ECC(S1.0%) were 9.06 nm, 9.07 nm, and 5.49 nm, respectively, the pore volume was the largest, and most of the pore structure was capillary and gel pores. When d > 1000 nm, the pore volume was almost zero, indicating that although there are some large pores, the pore depth was small. The pore structure distribution is shown in Figure 17e. The peak of each stage is the most permeable pore size in the aperture range; that is, the pore volume here was the largest, and the peak of CCCW-PE-ECC(X1.0%) in the range of pores and gel pores is less than that of PE-ECC and CCCW-PE-ECC(S1.0%), indicating that its maximum pore volume in each pore size range was small. The measured and predicted values of the total pore volume with pressure are shown in Figure 17f. When the pressure was close to 20,000 Pa, the pores in the material were almost filled with mercury. When the pressure exceeded 20,000 Pa, the total pore volume did not change much.

In summary, after having an appropriate amount of CCCW incorporated into it, the pore structure of PE-ECC was improved. Both the total pore volume and total pore area, or the porosity and permeability, demonstrated decreases. This is because, on the one hand, the CCCW can promote the cement hydration reaction, and the generated CaCO_3_, C-S-H gel, and calcium alumina (AFT) are beneficial for filling pores and cracks. On the other hand, the active substances in the CCCW can replace Ca^2+^ in unhydrated Ca(OH)_2_ in the cement and generate more stable C-S-H gel and CaCO_3_ crystals to fill capillaries and gel pores, improving the pore structure. The XYPEX-type CCCW more obviously improved the pore structure of the PE-ECC.

### 4.3. Micromorphology Test Analysis

**Figure 18:**
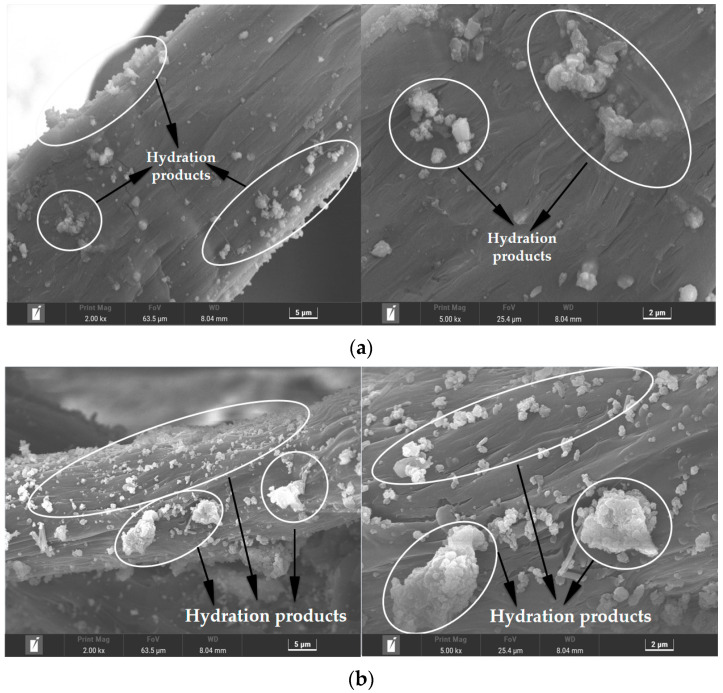

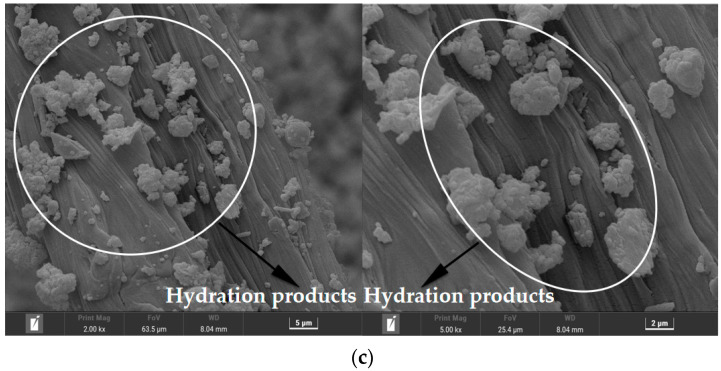
After chloride ion penetration: (**a**) PE-ECC; (**b**) CCCW-PE-ECC(X1.0%); (**c**) CCCW-PE-ECC(S1.0%).

The microscopic topographies of PE-ECC, CCCW-PE-ECC(X1.0%) and CCCW-PE-ECC(S1.0%) after chloride ion penetration are presented in Figure 18a–c, respectively. THe XRD results showed that the hydration products may be CaCO_3_, C-S-H, and AFT on the fiber surface. The amount and volume of hydration products on the surface of the PE-ECC fibers were decreased compared with CCCW-PE-ECC(X1.0%) and CCCW-PE-ECC(S1.0%). Hydration products on a fiber’s surface not only promote the connection between the fiber and the matrix but also fill the pores. Due to the reduction of hydration products, the pores originally filled by hydration products will reappear, resulting in the decrease of matrix compactness and the degradation of PE-ECC performance to a certain extent. The hydration products of CCCW-PE-ECC(X1.0%) and CCCW-PE-ECC(S1.0%) are greater in number and more dense than those of the PE-ECC after chloride ion permeation, indicating that they are more resistant to chloride ion attack than PE-ECC, which further indicates that the incorporation of a CCCW can effectively improve the chloride ion permeation resistance of PE-ECC.

## 5. Conclusions

To design cementitious composites with excellent mechanical and impermeability properties, the excellent tensile properties of PE-ECC and the unique impermeability properties of CCCW were combined. Different types and doses of CCCW were incorporated into the PE-ECC to study the effect of the CCCW on the mechanical and impermeability properties of the PE-ECC, and to analyze them at the microscopic level. The main conclusions are described as follows:

(1) With increasing CCCW doping, the mechanical properties of the PE-ECC tended to increase first and then decrease, and the mechanical properties were best when the doping amount was 1%. The mechanical properties of the PE-ECC were more obviously improved by the XYPEX-type CCCW, with a compressive strength of 53.8 MPa, flexural strength of 11.8 MPa, an ultimate tensile stress of 5.56 MPa, and an ultimate tensile strain of 7.53 MPa, which were 37.95%, 53.25%, 14.17%, and 21.65% higher than those of the reference, respectively.
(2) According to crack width meter and DIC analyses, the number of cracks in the middle region of the dog-bone specimens increased, the crack tolerance increased, the distribution was more uniform, and the crack control ability and tensile ductility were enhanced, after incorporating a suitable amount of the CCCW.
(3) With increased CCCW dosing, the seepage resistance of PE-ECC tended to increase and then decrease, and the best performance of PE-ECC was achieved when the dosing was 1%. CCCW-PE-ECC(X1.0%) and CCCW-PE-ECC(S1.0%) showed the smallest permeation heights, 2.6 mm and 2.8 mm, respectively, which are 69.77% and 68.18% lower than that of the baseline. The chloride ion diffusion coefficients of CCCW-PE-ECC(X1.0%) and CCCW-PE-ECC(S1.0%) exhibit the smallest values, 0.15 × 10^−12^ m^2^/s and 0.10 × 10^−12^ m^2^/s, respectively, which are 68.75% and 79.17% lower than that of the reference.
(4) Laser particle size distribution meter analysis showed that the XYPEX-type CCCW particle size was finer than that of SY1000. The XRD analysis showed that both CCCWs, with suitable doping, can enhance the C-S-H gel and CaCO_3_ intensity diffraction peaks of the PE-ECC and that the enhancement of the XYPEX-type CCCW was more obvious. The enhancement of the XYPEX-type CCCW on the PE-ECC against chloride ion permeation is mainly due to the generation of more hydration products to fill the pores and improve the structural compactness, while the SY1000-type CCCW mainly improves the performance against chloride ion permeation because it contains acrylamide and sodium fumarate, which perform an anti-corrosion function.
(5) MIP and SEM showed that the total pore volume, total pore area, permeability, and porosity of the PE-ECC decreased, and that the structure was more compact, after doping with two suitable doses of CCCW. The improvement in the pore structure of the PE-ECC was more obvious after doping with XYPEX-type CCCW. After doping with CCCW, the surface of the PE-ECC matrix was flatter, and the degree of erosion of hydration products on the PE fiber surface was reduced after chloride ion penetration.

## Figures and Tables

**Table 1:** Chemical compositions of the raw materials.

Ingredients (%)	SiO_2_	K_2_O	TiO_2_	Fe_2_O_3_	CaO	Al_2_O_3_	SO_3_
Cement	19.90	0.79	0.21	3.00	64.90	4.42	2.67
Fly ash	51.70	1.40	1.19	5.22	7.65	23.90	0.91

**Table 2:** Mix proportion (kg/m^3^) of PE-ECC CCCW-PE-ECC(X) and CCCW-PE-ECC(S).

	Sand	Cement	Fly Ash	Water	HRWR	Fiber	CCCW
PE-ECC	474.4	593.0	711.6	387.1	4.0	14.7	0
CCCW-PE-ECC(X0.5%)	474.4	593.0	711.6	387.1	4.0	14.7	6.52
CCCW-PE-ECC(X1.0%)	474.4	593.0	711.6	387.1	4.0	14.7	13.05
CCCW-PE-ECC(X1.5%)	474.4	593.0	711.6	387.1	4.0	14.7	19.57
CCCW-PE-ECC(X2.0%)	474.4	593.0	711.6	387.1	4.0	14.7	26.09
CCCW-PE-ECC(S0.5%)	474.4	593.0	711.6	387.1	4.0	14.7	6.52
CCCW-PE-ECC(S1.0%)	474.4	593.0	711.6	387.1	4.0	14.7	13.05
CCCW-PE-ECC(S1.5%)	474.4	593.0	711.6	387.1	4.0	14.7	19.57
CCCW-PE-ECC(S2.0%)	474.4	593.0	711.6	387.1	4.0	14.7	26.09

Note: PE-ECC is the reference group, CCCW-PE-ECC(X) indicates that the XYPEX-type CCCW is added. CCCW-PE-ECC(S) indicates that the SY1000-type CCCW is added. CCCW-PE-ECC(X0.5%) indicates that the XYPEX-type CCCW is added and its mass fraction is 0.5% of the cementitious material, CCCW-PE-ECC(S0.5%) indicates that the SY1000-type CCCW is added and its mass fraction is 0.5% of the cementitious material.

**Table 3:** MIP results of PE-ECC, CCCW-PE-ECC(X1.0%) and CCCW-PE-ECC(S1.0%) for 28 days.

	Total Intrusion Volume (mL/g)	Total Pore Area (m^2^/g)	Average Pore Diameter (nm)	Bulk Density (g/mL)	Apparent Density (g/mL)	Porosity (%)	Permeability (md)
PE-ECC	0.21	11.90	62.32	1.24	1.54	24.74	476.58
CCCW-PE-ECC(X1.0%)	0.14	9.43	47.54	1.49	2.16	19.32	122.34
CCCW-PE-ECC(S1.0%)	0.16	9.98	53.21	1.40	1.75	20.09	197.61

## Data Availability

The data presented in this study are available on request from the corresponding author upon reasonable request.
